# Genetic Analysis and Reproductive Intervention in a Duchenne Muscular Dystrophy Family With Cryptic Complex Chromosomal Rearrangement Identified by Structural Variation Sequencing

**DOI:** 10.1155/humu/8333541

**Published:** 2026-09-30

**Authors:** Shengfang Qin, Xueyan Wang, Jin Wang, Xiangyou Leng, Zhuo Zhang, Ximin Chen, Yan Yin

**Affiliations:** ^1^ Department of Medical Genetics and Prenatal Diagnosis Sichuan Provincial Women′s and Children′s Hospital/The Affiliated Women′s and Children′s Hospital of Chengdu Medical College Chengdu Sichuan China

**Keywords:** breakpoints, complex rearrangement, Duchenne muscular dystrophy, reproductive intervention, structural variant sequencing

## Abstract

Duchenne/Becker muscular dystrophy (DMD/BMD) is caused by variants in the X‐linked *DMD* gene—most commonly deletions, duplications, or point mutations, and only rarely by complex chromosomal rearrangements (CCRs). In this study, structural variant sequencing (SVseq) was applied to a 14‐year‐old male patient with DMD. Although the patient exhibited typical symptoms, conventional tests—including karyotype analysis, gene panel, whole‐exome sequencing (WES), and multiplex ligation–dependent probe amplification (MLPA)—were all negative. SVseq identified a novel complex rearrangement on the X chromosome, comprising a 974,456 bp inversion spanning the 5 ^′^ UTR and Exons 1–2 of the *DMD* gene, flanked by two microdeletions of 7285 and 6468 bp (NC_000023.11:g.[32865334_32872619del;32872619_33847075inv33847075_33853543del]). This rearrangement disrupts the *DMD* coding sequence and is classified as a pathogenic variant. Short tandem repeat (STR) analysis confirmed the familial relationship, whereas PCR and Sanger sequencing precisely mapped the breakpoints, establishing that the mutation arose de novo in his mother and was maternally inherited. Reproductive interventions—including preimplantation genetic testing for monogenic disorders (PGT‐M) and second‐trimester prenatal diagnosis—enabled the successful birth of a healthy male child. SVseq demonstrated clear advantages over standard methods for detecting CCRs, highlighting its value in genetically elusive cases. We recommend SVseq as a complementary diagnostic tool for unresolved DMD cases where conventional testing does not explain the findings. This approach can enhance diagnostic yield and support informed family planning.

## 1. Introduction

Dystrophinopathies, caused by pathogenic variants in the *DMD* gene, are among the best‐studied muscle diseases [1]. These disorders encompass Duchenne muscular dystrophy (DMD) (OMIM: 310200) and Becker muscular dystrophy (BMD) (OMIM: 300376). Dystrophinopathies primarily affect males, whereas females are usually asymptomatic carriers. DMD is the most common childhood muscular dystrophy, with an incidence of 1 in 3500–5000 live male births [2]. The main clinical manifestations of DMD include progressive, symmetrical muscle weakness, a positive Gowers′ sign, and pseudohypertrophy of the gastrocnemius muscle. After age 5, the disease progresses rapidly—most affected individuals lose the ability to walk independently by age 12. Cardiac or respiratory failure is the leading cause of death, usually occurring in the early twenties.

DMD and BMD are X‐linked myopathies caused by mutations in the *DMD* gene at Xp21, leading to deficient or abnormal dystrophin expression. The *DMD* gene spans approximately 2.4 Mb and contains 79 exons, encoding the 3685‐amino‐acid cytoskeletal protein dystrophin, which has a molecular weight of 427 kDa. To date, more than 7000 *DMD* gene variants have been reported. The majority are deletions (about 60%–75%), duplications (8%–15%), and small variants—including point mutations and small insertions or deletions (20%). Rarely, the *DMD* gene is disrupted by chromosomal rearrangements, which account for about 3% of all reported variants in the Human Gene Mutation Database (HGMD). These chromosomal rearrangements may include inversions, translocations, and complex structural variants [3–5].

Deletions and duplications in the *DMD* gene can be routinely detected using multiplex ligation–dependent probe amplification (MLPA) and, in some cases, chromosome microarray analysis (CMA) or copy number variant sequencing (CNVseq) [6, 7]. Currently, the most common approach for *DMD* gene analysis combines MLPA with targeted next‐generation sequencing (NGS) techniques, such as gene panels or whole‐exome sequencing (WES). This strategy enables the detection of various *DMD* gene variants—including point mutations, deletions, and duplications [8, 9]—and allows genetic diagnosis in more than 90% of suspected muscular dystrophy cases. However, these methods have notable limitations. Mutations at MLPA probe binding sites can result in false negatives. Conventional CNVseq may be hampered by insufficient sequencing depth, whereas CMA is limited by probe density and uneven distribution. Additionally, gene panels and WES primarily target exonic regions. Importantly, none of the above methods can detect balanced genomic rearrangements or precisely characterize balanced or unbalanced structural patterns [10–12], making it difficult to identify chromosomal inversions, balanced translocations, and complex rearrangements involving *DMD* [13, 14].

Structural variant sequencing (SVseq) technology, which uses mate‐pair libraries, enables high‐throughput sequencing for the analysis of chromosomal structural variations (SVs) [15, 16]. This approach can detect structural changes larger than 100 kb, including deletions, duplications, translocations, inversions, insertions, and complex rearrangements [17, 18]. In this study, SVseq was used to identify rearrangements in the *DMD* gene of a patient clinically diagnosed with DMD, whose condition could not be adequately explained by conventional methods such as MLPA, WES, or a targeted *DMD* gene panel. By implementing SVseq, we aim to clarify the genetic basis of the patient′s disease, provide a robust foundation for diagnosis and treatment, and help prevent the transmission of the genetic disorder in future pregnancies.

## 2. Materials and Methods

### 2.1. Subjects

The parents sought assistance at the reproductive center for assisted reproduction, reporting that their 14‐year‐old son had been diagnosed with DMD. The boy was unable to walk and could not undergo a clinical examination at our hospital. According to his parents, he was wheelchair‐dependent, exhibited muscle atrophy in his limbs, and had joint stiffness. After physical activity, he experienced shortness of breath, wheezing, frequent fatigue, and occasional palpitations (no electrocardiogram was performed). He often developed shortness of breath after daily activities and had episodes of choking and coughing when eating or drinking.

The child was born at full term in 2010. His developmental milestones were delayed: He could not lift his head at 3 months or crawl at 9 months. He began walking at 1.5 years of age, but his gait was unstable, and he frequently fell. At age 2, he was clinically diagnosed with DMD at another hospital. By age 4, he displayed typical symptoms, including dystonia, proximal muscle weakness, difficulty raising his upper limbs, difficulty climbing stairs and squatting, a waddling (duck) gait, and a positive Gowers sign. The pseudohypertrophy was characterized by a thick, hard, and weak texture. At age 11, although in 5th grade, he became unable to walk and subsequently dropped out of school.

Laboratory tests of his 5‐year‐old showed markedly elevated serum creatine kinase (CK) and abnormal muscle enzyme levels: CK 6623.80 U/L (reference: 40–200), CK‐MB 413.55 U/L (reference: 0–24), and CK‐MB/CK 6.24% (reference: < 5%). Immunohistochemical staining for dystrophin in two muscle biopsies supported the diagnosis of DMD. Muscle tissue from the right gastrocnemius (reddish‐brown in color) was used for histopathological analysis. Gomori trichrome staining revealed muscle fibers ranging in diameter from approximately 10 to 65 *μ*m, with a heterogeneous distribution. Focal degeneration, necrosis, and regenerating fibers were observed, with no definite lymphocyte aggregation or infiltration and only mild interstitial fibrosis. NADH‐TR staining demonstrated enzyme activity, either concentrated or absent, at sites of degeneration and necrosis. PAS staining showed variable glycogen content in focal muscle fibers. Oil Red O staining revealed mildly increased lipid content in focal areas. Acid phosphatase staining indicated increased enzyme activity at sites of degeneration and necrosis, in small blood vessels, and with mild interstitial fibrosis. ATPase staining revealed two types of muscle fibers. Immunohistochemistry revealed that antibody staining for dystrophin (NRC)—including the N‐terminal, rod domain, and C‐terminal—was either completely or nearly absent. In contrast, the normal control group exhibited clear dystrophin localization on the sarcolemma, with complete circular positive expression. Meanwhile, staining for dysferlin and sarcoglycan *α*, *β*, *γ*, and *δ* remained positive.

Despite extensive testing at several hospitals—including chromosome karyotype analysis, *DMD* gene testing, MLPA, and WES—no pathogenic *DMD* gene variants were identified. The mother had previously terminated four pregnancies for unrelated reasons, with the sex of the fetuses unknown. In 2020, she conceived naturally and underwent prenatal diagnosis at another hospital at 17 weeks of gestation. The fetus was confirmed to be male, and the pregnancy was electively terminated based on gender selection. Additional information can be found in the pedigree diagram (Figure 1).

**Figure 1:**
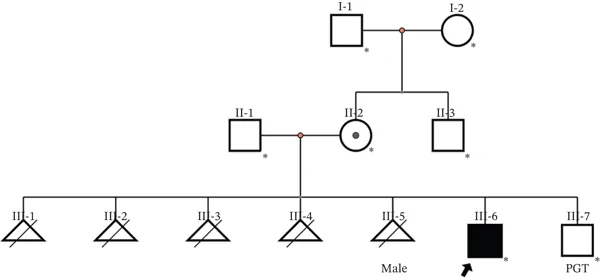
Family diagram. I, II, and III: generation number; 1, 2, and 3: individual number; unfilled symbol: unaffected; half‐filled symbol: carrier; filled symbol: affected; arrow: proband; small triangle with slash: spontaneous abortion; PGT: preimplantation genetic testing; ∗: genetic testing.

This study was approved by the Institutional Review Board of Sichuan Women and Children′s Hospital/Chengdu Medical College Affiliated Women and Children′s Hospital (approval number: 20240822‐345). Written informed consent was obtained from all participants.

### 2.2. Specimen Preparation and DNA Extraction

A volume of 2 mL of venous blood was collected from the patient and his family members. Genomic DNA was extracted from EDTA‐Na2 anticoagulated blood using the DNeasy Blood and Tissue Kit (QIAGEN), following the manufacturer′s protocol. DNA quality was considered acceptable if the concentration exceeded 30 ng/*μ*L and the OD260/OD280 ratio was between 1.8 and 2.0, as determined by a NanoDrop 1C ultraviolet spectrophotometer (Thermo Fisher Scientific). The study was approved by the Institutional Review Board, and informed consent was obtained from all participants prior to molecular analysis.

### 2.3. SVseq Analysis

SVseq detection utilizes the MP library kit (Genentech Co., Ltd., Shanghai, China), based on the mate‐pair sequencing strategy, to capture large genomic fragments exceeding 100 kb. Genomic DNA is randomly fragmented to a set size (2–8 kb, typically ~5 kb), followed by ligation, removal of nonligated fragments, further fragmentation, magnetic bead enrichment, purification of biotin‐labeled fragments, end repair, A‐tailing, adapter ligation, and PCR amplification. The ends of these DNA fragments are sequenced on the DNBSEQ‐T7 platform in paired‐end 150 bp mode, achieving > 3× base coverage (i.e., the number of times each base was sequenced), calculated by (mapped reads × read length)/genome size. The raw data size is at least 15 GB, with Q20 > 90*%*, Q30 > 85*%*, and mapping rate > 80*%*. After sequencing, adapter sequences are removed, and reads are quality‐filtered with FastP. High‐quality paired reads are aligned to the NCBI human reference genome (GRCh38) using BWA, and only uniquely mapped reads are retained for further analysis [15, 17, 18]. Sequencing data are rigorously filtered: Abnormally mapped paired reads (chimeric pairs) are extracted to detect clusters indicating SVs. Systematic errors are minimized using a population‐based SV dataset to filter out common false positives arising from technical or population differences. Random errors are filtered based on sequence cluster characteristics, such as read count. Authentic SVs—including translocations, inversions, and insertions—are further validated using alignment chain information. Both copy number variations (CNVs) and SVs are analyzed using paired‐read approaches: CNVs are identified with FreeC, whereas SVs are detected with custom software considering insert size and read orientation to flag abnormal mapping. The direction and distance of reads provide supporting evidence for structural variants. Breakpoint signals, as seen in deletions, duplications, or inversions, confirm chromosomal changes (see Figure 2). Sanger sequencing is used to determine the base sequence at identified breakpoints. SVseq can robustly detect chromosomal structural changes, including complex rearrangements, by integrating signals from translocations, deletions, duplications, and inversions. Mate‐pair read coverage refers to bridged coverage, a concept unique to mate‐pair sequencing. Each mate‐pair read consists of two segments from the ends of the same large fragment; their presence indicates that the entire region between the two segments—the “bridge”—is covered. The number of such “bridges” (i.e., mate‐pair reads) spanning each genomic base is reported as the bridged coverage value.

**Figure 2:**
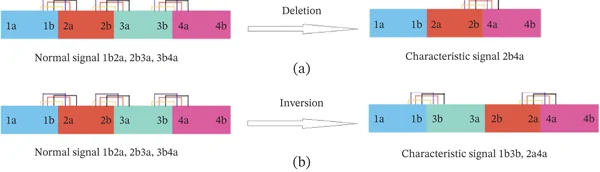
SVseq identifies chromosomal structural variations, including deletions and inversions, by detecting characteristic signals. The normal connection signals among the four fragments CNV1‐4 are 1b2a, 2b3a, and 3b4a. (a) Detection of the deletion‐specific signal 2b4a indicates a deletion of CNV3. (b) When the deletion signals 1b3b and 2a4a are observed, this suggests an inversion has occurred between CNV3 and CNV2.

### 2.4. Short Tandem Repeat (STR) Analysis and Family Relationship Confirmation

Family relationships were assessed using STR sequence analysis. STR genotyping was performed using the Goldeneye DNA Identification System 20A (Peoplespot, Beijing) to target 20 STR loci. The standard PCR amplification reaction had a total volume of 25 *μ*L: deionized water, 7 *μ*L; 2.5× PCR reaction buffer II, 10 *μ*L; 5× 20A primer mixture, 5 *μ*L; Taq polymerase II, 0.5 *μ*L; and template DNA, 2.5 *μ*L (approximately 0.25–4.0 ng). The PCR conditions were 95°C for 5 min; 30 cycles of 94°C for 30 s, 60°C for 1 min, and 70°C for 1 min; followed by a final extension at 60°C for 30 min. Amplified products were analyzed by capillary electrophoresis on an AB 3500Dx genetic analyzer, and data were processed with GeneMapper software.

### 2.5. Sanger Sequencing Confirms Breakpoint Sequence

Specific breakpoints of complex rearrangements identified by SVseq were validated using conventional PCR followed by Sanger sequencing. PCR amplification was performed with the 2× T2xPlus Master Mix P211 protocol (Vazyme, Nanjing, China). For Junction 1, primers AGGGCATTTAGTGTAGCCATTTCT (forward) and TTGCTCCCACTTTTGACATTCCAT (reverse) were used. For Junction 2, primers CTGATGGTGAGGTGGTAGCC (forward) and ATCTGGAGGTACACCCAATTAGT (reverse) were used. The PCR conditions were 93°C for 3 min; 35 cycles of 95°C for 30 s, 60°C for 30 s, and 72°C for 1 min; followed by a final extension at 72°C for 5 min. PCR products were analyzed by electrophoresis and sequenced using the ABI 3500XL Dx Genetic Analyzer (Thermo Fisher Scientific, Waltham, Massachusetts, United States). Sequence data were processed using CodonCode Aligner (CodonCode Corporation, Centerville, Utah, United States) and aligned to the GRCh38/hg38 reference genome.

### 2.6. Preimplantation Genetic Testing and Prenatal Diagnosis

The parents chose preimplantation genetic testing when planning to conceive their second child. Preimplantation genetic testing for monogenic disorders (PGT‐M) was performed, which is suitable for detecting rearrangement fragments smaller than 1 Mb. Initially, a haplotype was constructed using genomic DNA from the parents and their affected child. Subsequently, 2 million heterozygous single‐nucleotide polymorphism (SNP) sites in the *DMD* gene region and chromosomal aneuploidies were analyzed in cultured blastocysts. SNP loci near the *DMD* gene breakpoints were assessed using high‐throughput DNA sequencing, a proprietary detection method (mapping allele with resolved carrier status [MARECS]), the PGXCloud system 2.7.5, and PGD analysis software 2.0. One normal embryo was selected and transferred into the uterus. Prenatal diagnosis in the second trimester confirmed the absence of *DMD* gene variants by Sanger sequencing.

## 3. Results

### 3.1. SVseq Results

The SVseq sequencing data demonstrated high quality. The raw data size was 33.35 GB, with 140,229,989 reads aligned after deduplication. Clean sequence proportions with Q20 and Q30 scores were 94.34% and 85.50%, respectively, and the alignment rate reached 99.44%. The average base coverage depth was 5.6×. Mate‐pair reads represent SV‐supporting read pairs. The direct evidence for two breakpoint junctions is provided by mate‐pair reads: 34 pairs and 25 pairs, respectively, corresponding to bridged coverages of 34× and 25×.

SVseq analysis revealed a complex chromosomal rearrangement (CCR) on the X chromosome, comprising a large inversion and two flanking deletions. In the Xp21.1 region, a 974,456 bp inverted segment was identified, spanning the *DMD* gene 5 ^′^ UTR and Exons 1–2. Two small deleted fragments, measuring 7285 and 6468 bp, border the inversion (NC_000023.11:g.[32865334_32872619del;32872619_33847075inv;33847075_33853543del]). Notably, the 6468 bp deletion resides within the second intron of the *DMD* gene (see Figures 3 and 4).

**Figure 3:**
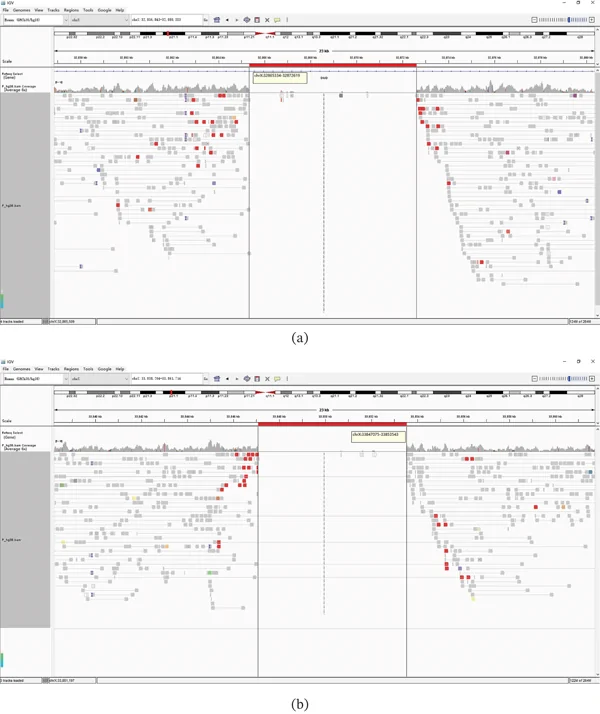
Visualization of the BAM file from SVseq for the patient using the Integrated Genomics Viewer (IGV). (a,b) IGV displays the breakpoints, along with two small deletion fragments measuring 7285 and 6468 bp, located at chrX:32865334–32872619 (within the second intron of the *DMD* gene) and chrX:33847075–33853543, respectively, as illustrated. Sequence reads are absent in the deleted regions, highlighted in red bars, and the exact intervals are shown in the boxes with a yellow background.

**Figure 4:**
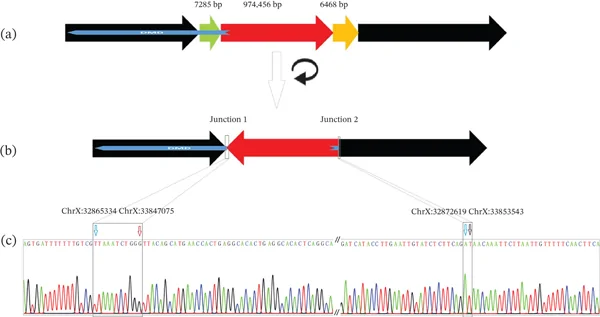
SVseq results for X chromosome complex rearrangements. (a) The normal X chromosomes. (b) The rearranged X chromosome in the patient. The Xp21.1 region exhibits an inversion fragment measuring 974,456 bp (chrX:32872619–33847075), encompassing the 5 ^′^ UTR and Exons 1–2 of the *DMD* gene. Additionally, two small deletion fragments of 7285 and 6468 bp (chrX:32865334–32872619 and chrX:33847075–33853543, respectively) were identified flanking the inversion region. Notably, the 6468 bp fragment (chrX:33847075–33853543) is located within Intron 2 of the *DMD* gene. (c) The two breakpoint sequences on the X chromosome were confirmed by Sanger sequencing. Junction 1 is characterized by a 9‐base insertion between chrX:32865334 and chrX:33847075. Junction 2 shows a direct connection between chrX:32872619 and chrX:33853543, shown as the grey boxes.

### 3.2. Family Relationship Confirmation

A total of 20 STR loci were analyzed, all of which conformed to Mendelian inheritance patterns, confirming that the alleles in the children were inherited from both parents. Integrating family pedigree information and DNA analysis further validated the biological relationships among the mother, uncle, grandparents, and the patient′s parents, as shown in Figure 5.

**Figure 5:**
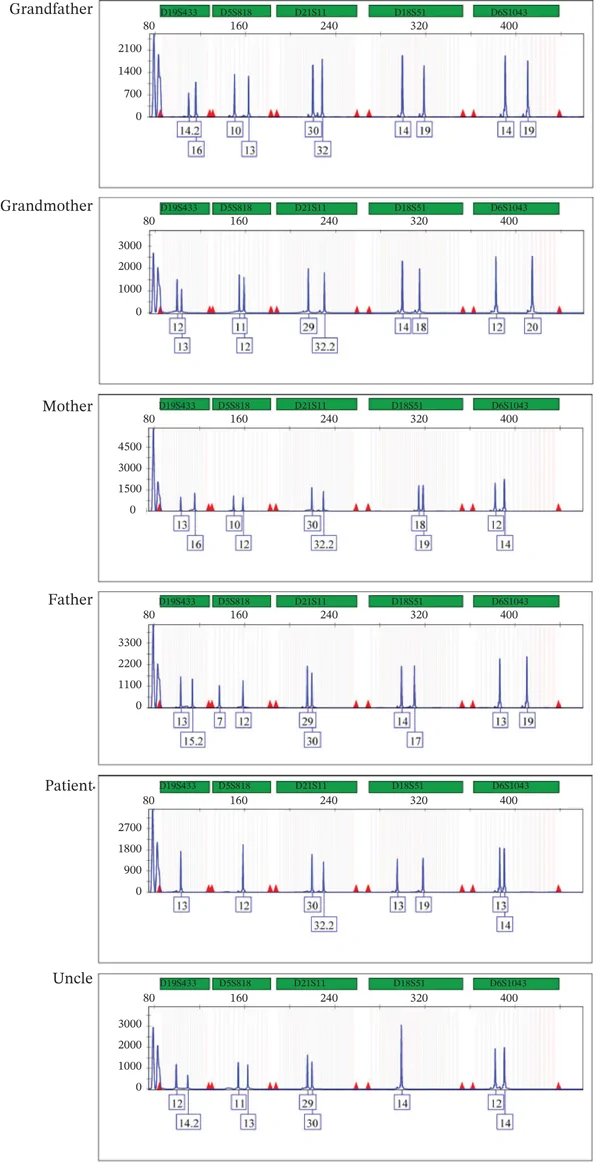
Confirmation of family relationships. Relationships among family members were confirmed by comparing 20 STR loci. The figure displays five representative STR loci (D19S433, D5S818, D21S11, D18S51, and D6S1043) for each family member. A total of 20 STR loci were tested, all of which followed Mendelian inheritance patterns—meaning the alleles of the children could be attributed to both parents. Combined analysis of the family pedigree and DNA results confirmed the biological relationships among the mother, uncle, grandparents, and between the patient and his parents.

### 3.3. Confirmation of Breakpoints Through Sanger Sequencing

PCR amplification with specific primers revealed that both the child and the mother carried the same *DMD* gene variant, whereas other family members were wild‐type (see Figure 6). Family analysis confirmed the mother as a carrier of this novel *DMD* variant. Sanger sequencing indicated that the base sequence at the first inverted breakpoint junction was chrX:32865334‐TAAATCTGG‐chrX:33847075, and the second junction directly connected chrX:32872619 and chrX:33853543 (see Figure 4c).

**Figure 6:**
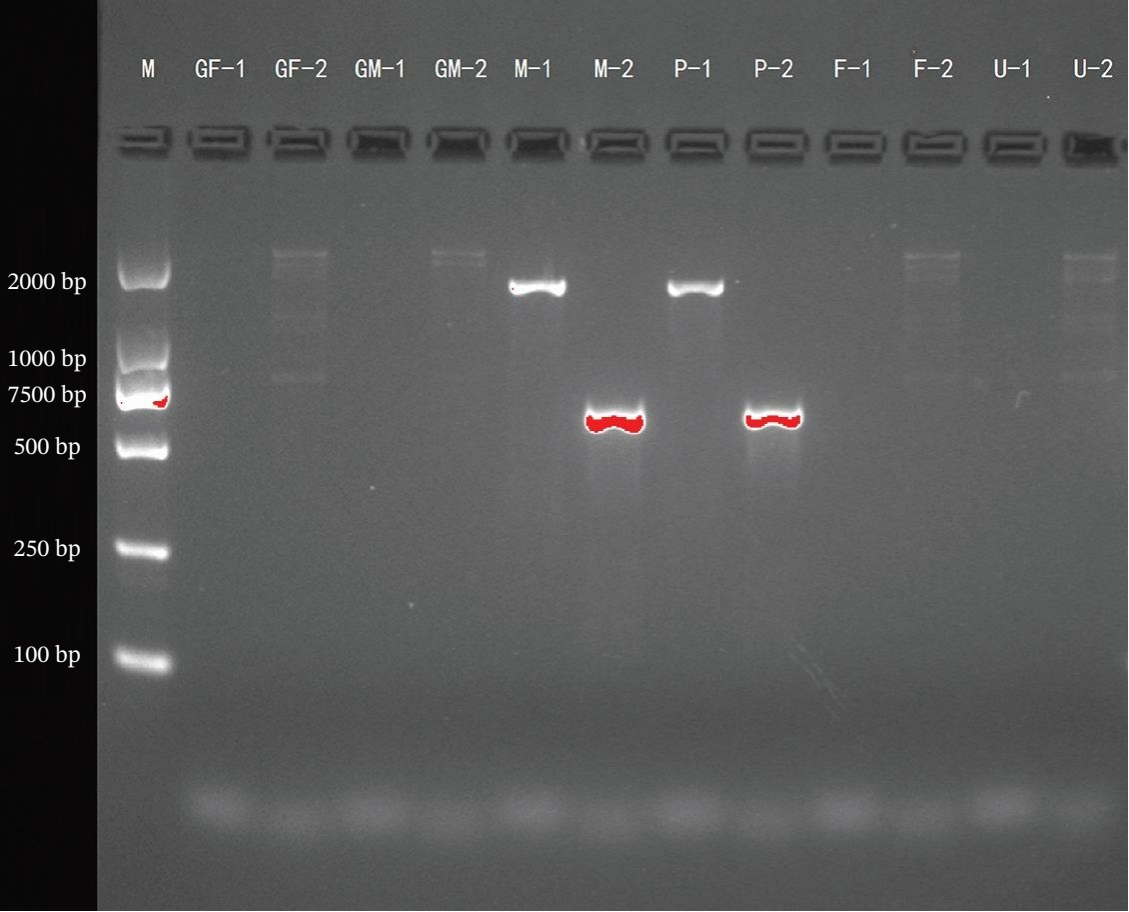
Electrophoretic analysis of *DMD* inversion. PCR amplification was performed using inversion‐specific primers. Electrophoresis results showed that the mother and the patient had identical fragments, indicating *DMD* inversion, whereas the other family members displayed a wild‐type pattern. GF: grandfather; GM: grandmother; M: mother; F: father; P: patient; U: uncle; 1: Primer 1; 2: Primer 2.

### 3.4. Blockage of DMD in Family Reproduction

A haplotype was constructed using genetic data from the parents and the proband, focusing on SNP sites located 2 Mb upstream and downstream of the *DMD* gene. Four blastocysts were developed, three of which were euploid. Among the euploid embryos, two did not carry the pathogenic *DMD* variant. A normal embryo was selected and implanted into the mother′s uterus, leading to a successful pregnancy. Prenatal diagnosis confirmed that the fetus did not carry the pathogenic *DMD* variant. The mother delivered a healthy boy at term, and follow‐up evaluations at 1 year indicated normal development. Comprehensive clinical assessments by the pediatric department revealed no abnormalities, including normal language and motor milestones. The infant began walking at 11 months. At 12 months, peripheral blood samples were collected, and Sanger sequencing verified the absence of the *DMD* variant using breakpoint PCR amplification and electrophoresis. No variant identical to the proband was detected, and CK levels in venous blood were within the normal range.

## 4. Discussion

Detecting *DMD* gene variants presents significant challenges due to the gene′s large size and the wide spectrum of mutations contributing to its instability [19]. Multiple analytical methods are routinely employed in clinical practice to detect *DMD* gene variants, including MLPA, CMA, CNVseq, WES, targeted panels, long‐range PCR, and Sanger sequencing. Consequently, a combination of several methods is often required. Commonly, two, three, or even four techniques are used together, such as MLPA with targeted NGS [8]; MLPA combined with PCR, microarray, and whole‐genome sequencing (WGS) [20]; WES and optical genome mapping (OGM) with long‐read sequencing [21–23]; or WGS with Sanger sequencing [5]. In recent years, emerging technologies have been gradually incorporated, such as CNV and breakpoint data analysis via NGS and WGS [24], OGM [22, 25], and long‐read sequencing [23, 26–28]. However, conventional methods have well‐recognized limitations in detecting chromosomal SVs. For instance, CMA/CNVseq can only identify unbalanced CNVs, whereas chromosome karyotyping, due to its limited resolution, can only detect large‐scale SVs (> 5 Mb). Standard 30× WGS has a relatively low detection rate for balanced rearrangements and complex SVs. The OGM is limited by the number of restriction enzyme sites and the need for specialized equipment. Theoretically, high‐depth, third‐generation WGS can comprehensively detect all *DMD* gene variant types, including balanced chromosomal rearrangements (BCR), inversions, and CCR. Nevertheless, high sequencing costs and the complexity of data analysis currently restrict its widespread clinical adoption.

To facilitate *DMD* gene rearrangement detection and streamline clinical workflows, this study employed SVseq technology for clinically diagnosed DMD patients whose variants could not be identified by conventional methods such as MLPA and NGS. SVseq can accurately pinpoint breakpoints within 1000 base pairs and define them in a single PCR run followed by Sanger sequencing. Our previous research [29], as well as studies from other groups [15–18], has demonstrated that SVseq provides significant advantages for detecting SVs, particularly BCR, inversions, and CCR. SVseq offers a high detection rate, rapid turnaround, lower costs, simple methodology, broad specimen compatibility, and strong clinical applicability. We, therefore, recommend SVseq as a complementary option for some unresolved DMD cases by conventional testing.

The results of this study indicate that the patient harbors a maternally inherited inversion of the *DMD* gene (chrX:g.[32865334_32872619del;32872619_33847075inv;33847075_33853543del]). Loss of function (LOF) is the established pathogenic mechanism for the *DMD* gene. When the 5 ^′^ UTR and Exons 1–2 regions are inverted, the MANE transcript (NM_004006.3) becomes structurally incomplete, preventing efficient translation of the DMD protein in muscle tissue. Although several shorter *DMD* transcripts exist, most exhibit very low or negligible expression in muscle. This study did not investigate the RNA or protein‐level effects of this variant. However, the patient is a hemizygote for an X‐linked recessive disorder and presents with a highly specific DMD clinical phenotype, supporting the conclusion that this variant is expected to impair *DMD* gene function. Furthermore, this variant is absent from the gnomAD population database. According to American College of Medical Genetics and Genomics (ACMG) guidelines [30], this variant is classified as pathogenic (PVS1 + PM2 + PM6). In the future, we plan to conduct functional analyses to further characterize the impact of this SV, once patient samples and informed consent are obtained.

The genetic diagnosis of the patient subsequently guided the family′s reproductive decisions. For the small fragment deletion and inversion in the *DMD* gene, the PGT‐M protocol was used for assisted reproduction. A haplotype was first constructed using peripheral blood samples from the parents and the proband, focusing on specific SNP sites within a 2 Mb region flanking the *DMD* variant. Cultured blastocysts were tested for chromosomal aneuploidy and the target *DMD* variant. Ultimately, one unaffected embryo was selected for transfer. Prenatal diagnosis in the second trimester was negative for the pathogenic variant, the pregnancy proceeded to term, and follow‐up assessments 1 year after birth indicated normal development. This study identified the genetic cause of the patient′s condition, offered a reproductive intervention strategy for the family, and brought closure to a decade‐long period of uncertainty for the parents, who had previously been reluctant to expand their family due to the unresolved genetic issue.

Pseudomuscular dystrophy, particularly DMD, carries a high fatality rate and poor prognosis. Currently, no effective treatment exists, placing a significant burden on affected families and society. To help prevent the birth of children with DMD, genetic counseling is essential for families with a history of the disease. Additionally, PGT and prenatal diagnosis—once the pathogenic gene and variant type in the proband are identified—are effective strategies to prevent disease recurrence. Approximately two‐thirds of *DMD* variants are inherited, whereas de novo mutations account for up to one‐third. The high rate of de novo mutations in *DMD*—about 33%—may explain the elevated incidence in families with no prior history [31, 32]. Therefore, even families without a reproductive or family history of DMD should be aware of the potential risk. In cases involving de novo mutations, prenatal screening for pathogenic *DMD* variants can be performed using maternal peripheral blood samples to analyze fetal cell‐free DNA during the first and second trimesters. This approach enables prenatal diagnosis of high‐risk fetuses, effectively preventing and controlling the occurrence of DMD.

## Funding

This study was funded by the Affiliated Women′s and Children′s Hospital of Chengdu Medical College, Clinical Science Research, 24LHFYSZ1‐20.

## Conflicts of Interest

The authors declare no conflicts of interest.

## Data Availability

The datasets used and/or analyzed during the current study are available from the corresponding author on reasonable request.
